# Factors associated with favourable neurological outcomes following cardiopulmonary resuscitation for out-of-hospital cardiac arrest: A retrospective multi-centre cohort study

**DOI:** 10.1016/j.resplu.2024.100574

**Published:** 2024-02-08

**Authors:** Naoki Tominaga, Toru Takiguchi, Tomohisa Seki, Takuro Hamaguchi, Jun Nakata, Takeshi Yamamoto, Takashi Tagami, Akihiko Inoue, Toru Hifumi, Tetsuya Sakamoto, Yasuhiro Kuroda, Shoji Yokobori, SAVE-J II study group Investigation Supervision

**Affiliations:** a Department of Emergency and Critical Care Medicine, Nippon Medical School, Tokyo, Japan; b Department of Healthcare Information Management, The University of Tokyo Hospital, Tokyo, Japan; c Division of Cardiovascular Intensive Care, Department of Cardiovascular Medicine, Nippon Medical School Hospital, Tokyo, Japan; d Department of Emergency and Critical Care Medicine, Hyogo Emergency Medical Centre, Kobe, Japan; e Department of Emergency and Critical Care Medicine, St. Luke’s International Hospital, Tokyo, Japan; f Department of Emergency Medicine, Teikyo University School of Medicine, Tokyo, Japan; g Department of Emergency Medicine, Kagawa University School of Medicine, Kagawa, Japan

**Keywords:** Extracorporeal cardiopulmonary resuscitation, Neurological outcome, Out-of-hospital cardiac arrest, Pupillary reflex, Body movement, Gasping

## Abstract

**Aim:**

To investigate the factors associated with favourable neurological outcomes in adult patients undergoing extracorporeal cardiopulmonary resuscitation (ECPR) for out-of-hospital cardiac arrest (OHCA).

**Methods:**

This retrospective observational study used secondary analysis of the SAVE-J II multicentre registry data from 36 institutions in Japan. Between 2013 and 2018, 2157 patients with OHCA who underwent ECPR were enrolled in SAVE-J II. A total of 1823 patients met the study inclusion criteria. Adult patients (aged ≥ 18 years) with OHCA, who underwent ECPR before admission to the intensive care unit, were included in our secondary analysis. The primary outcome was a favourable neurological outcome at hospital discharge, defined as a Cerebral Performance Category score of 1 or 2. We used a multivariate logistic regression model to examine the association between factors measured at the incident scene or upon hospital arrival and favourable neurological outcomes.

**Results:**

Multivariable analysis revealed that shockable rhythm at the scene [odds ratio (OR); 2.11; 95% confidence interval (CI), 1.16–3.95] and upon hospital arrival (OR 2.59; 95% CI 1.60–4.30), bystander CPR (OR 1.63; 95% CI 1.03–1.88), body movement during resuscitation (OR 7.10; 95% CI 1.79–32.90), gasping (OR 4.33; 95% CI 2.57–7.28), pupillary reflex on arrival (OR 2.93; 95% CI 1.73–4.95), and male sex (OR 0.43; 95% CI 0.24–0.75) significantly correlated with neurological outcomes.

**Conclusions:**

Shockable rhythm, bystander CPR, body movement during resuscitation, gasping, pupillary reflex, and sex were associated with favourable neurological outcomes in patients with OHCA treated with ECPR.

## Highlights

• This retrospective observational study aimed to identify predictive factors associated with favorable neurological outcomes using the data from a retrospective multicenter observational study (SAVE-J II) in patients with out-of-hospital cardiac arrest undergoing extracorporeal cardiopulmonary resuscitation.
• Shockable rhythm, bystander CPR, body movement during resuscitation, gasping, pupillary reflex, and sex were associated with favourable neurological outcomes in patients with out-of-hospital cardiac arrest undergoing extracorporeal cardiopulmonary resuscitation.
• Our findings may support the patient selection to administer extracorporeal cardiopulmonary resuscitation.

## Introduction

Extracorporeal cardiopulmonary resuscitation (ECPR) has been reported to improve outcomes in patients with out-of-hospital cardiac arrest (OHCA), compared with conventional cardiopulmonary resuscitation (CCPR).^1^, ^2^, ^3^, ^4^ Three randomised controlled trials of ECPR for OHCA recently reported controversial results.^5^, ^6^, ^7^ The ARREST trial was terminated early due to the superiority of ECPR over CCPR.^5^ However, the Prague OHCA and INCEPTION trials demonstrated no significant improvement in survival, with neurologically favourable outcomes.^6^, ^7^ Because ECPR has a higher socioeconomic cost than that of CCPR, patient selection must be appropriate.^8^

Previous guidelines and studies have suggested the selection criteria for the use of ECPR.^9^, ^10^ However, these guidelines also indicate a lack of robust data to identify the potential beneficiaries of ECPR.^9^ Several predictive factors for favourable neurological outcomes have been reported as necessary additions to the inclusion criteria for ECPR. These factors include age, initial shockable rhythm, witness status, and the duration of low blood flow.^11^, ^12^, ^13^, ^14^, ^15^, ^16^, ^17^, ^18^, ^19^, ^20^ However, the number of reported cases is small, with at most 500 in one study and overall less than 1000 in systematic reviews.^12^, ^18^, ^20^ Therefore, no consensus has been reached and the selection criteria for ECPR have not been established.^9^

This study aimed to identify predictive factors associated with favourable neurological outcomes using the data from a retrospective multicentre observational study called the Study of Advanced Life Support for Ventricular Fibrillation with Extracorporeal Circulation in Japan (SAVE-J II). Identifying these factors is crucial for establishing patient selection criteria and standardized protocols for ECPR.

## Materials and methods

### Ethical approval and consent to participate

The SAVE-J II study was registered in the University Hospital Medical Information Network Clinical Trials Registry and the Japanese Clinical Trial Registry (registration number: UMIN000036490). This study was approved by the Institutional Review Board of Kagawa University (approval number: 2018–110) and each participating institution, including the Nippon Medical School Hospital (approval number: R1-05–1125). The study design was approved by the Institutional Review Board of the Nippon Medical School Hospital (approval number: B-2022–633). The requirement for patient consent was waived at all participating institutions because of the retrospective nature of this study. This study adhered to the Strengthening the Reporting of Observational Studies in Epidemiology (STROBE) statement.

### Study design

This study was a secondary analysis of the SAVE-J II study data, which was a retrospective multicentre registry of patients with OHCA who had undergone ECPR within 36 participating institutions in Japan from 1 January 2013 to 31 December 2018.^21^

### Study sample

The inclusion criteria were: adult patients (age ≥ 18 years) with OHCA treated with ECPR before intensive care unit (ICU) admission. The exclusion criteria were: (1) implementation of veno-arterial extracorporeal membrane oxygenation (ECMO) after ICU admission; (2) procedure aborted after cannulation and before ECMO initiation because of the return of spontaneous circulation (ROSC); (3) cardiopulmonary arrest from external causes such as hypoxia, drug intoxication, trauma, suffocation, drowning, and other external causes; (4) patients achieving ROSC at hospital arrival or initiation of ECMO; (5) patients transferred from another hospital; and (6) patients with unknown neurological outcomes. Hypothermia was defined as a body temperature below 30 °C or as diagnosed by a physician at hospital admission.^21^

### Data collection

The SAVE-J II study database was compiled retrospectively through a comprehensive review of all hospital-based data from each participating institution, adhering to a reporting format specified by the lead investigator of the SAVE-J II study. Data collection was guided by the patients' medical histories and examinations. The data were registered at the discretion of the treating physicians and medical staff at each participating institution. In instances of uncertainty or abnormal values in the database, the principal investigators proactively contacted the respective facilities directly through electronic mail, phone calls, web conferences, or in-person meetings to verify the information. The characteristics of the participating institutions, including the inclusion and exclusion criteria, have been previously described.^22^ The following patient data were collected from the SAVE-J II database: age, sex, aetiology of cardiac arrest, initial cardiac rhythm at the scene and on hospital arrival, incidence of witnessed cardiac arrest and bystander-initiated cardiopulmonary resuscitation (CPR), use of defibrillation, ROSC, incidence of gasping on arrival, pupil diameter, pupillary reflex on arrival, blood gas analysis on arrival (such as pH, blood oxygen and carbon dioxide levels, lactate, and haemoglobin), cardiac rhythm before ECMO initiation, time interval information related to ECPR. Initial shockable rhythm was defined as ventricular fibrillation, pulseless ventricular tachycardia, or rhythm for defibrillation in automated external defibrillator used by emergency medical staff.^21^ ROSC was defined as at least one minute of continuing confirmation of pulsation.^21^ Transient ROSC was defined as a palpable pulse or measurable blood pressure before arrival at the hospital, with the patient in cardiac arrest on hospital arrival.^23^ Body movement during resuscitation was defined as having a Glasgow Coma Scale-Motor Score ≥ 2 in patients with cardiac arrest upon hospital arrival.^24^ Data such as the lengths of hospital and ICU stays, in-hospital mortality, and neurological outcomes were also collected. Time intervals were calculated as follows: (1) from the emergency medical services call to hospital arrival, (2) from hospital arrival to ECMO initiation, and (3) from the emergency medical services call to ECMO initiation. The definition of estimated low-flow time depended on the location of the cardiac arrest. If the cardiac arrest occurred in the ambulance, low-flow time was defined as the time from cardiac arrest to ECMO initiation. If the cardiac arrest occurred anywhere other than an ambulance, low-flow time was defined as the time from the emergency medical services call to ECMO initiation.^21^ We suspected multicollinearity when examining the variance inflation factor of the time intervals (i.e., from the emergency medical services call to hospital arrival, from hospital arrival to ECMO initiation, from the emergency medical services call to ECMO initiation, and estimated low-flow time); therefore, we only included the estimated low-flow time in our analysis. Similarly, a correlation with initial shockable rhythm at the scene and defibrillation could lead to collinearity in clinical situations. Therefore, we excluded defibrillation from the revised analyses.

### Outcome

The primary outcome was a favourable neurological outcome at the time of hospital discharge, assessed using the cerebral performance category (CPC) scale.^25^ A favourable outcome was defined as a CPC score of 1 or 2, whereas an unfavourable outcome was defined as a CPC score of 3, 4, or 5.

### Statistical analysis

Descriptive statistics were used to summarize baseline characteristics and outcomes during ECPR. Continuous variables were presented as median values and interquartile ranges, whereas categorical variables were presented as numbers and percentages. We compared neurological outcomes using the Mann-Whitney U test for continuous variables and Fisher’s exact or chi-squared tests for categorical variables. Univariate and multivariate logistic regression analyses were performed to determine favourable neurological outcomes. The explanatory variables used in the logistic regression analysis included those available to clinicians in the time period between initial point-of-contact with the patient to ECMO initiation. In the multivariable models, we adjusted for age, sex, aetiology of arrest (cardiogenic vs. non-cardiogenic), initial cardiac rhythm at the scene and on hospital arrival (shockable rhythm vs. non-shockable rhythm), witness-status of the cardiac arrest, bystander-initiated CPR, transient ROSC, estimated low-flow time, body movement during resuscitation, gasping on arrival, pupil diameter and pupillary reflex on arrival, and blood gas analysis on arrival. Previous studies have shown that these are favourable prognostic factors for OHCA treated with ECPR.^1^, ^10^, ^11^, ^13^, ^14^, ^15^, ^16^, ^17^, ^18^, ^19^ We selected clinically significant results for the ‘Results’ section and further discussed those with statistical significance (odds ratio > 2, <0.5) in the ‘Discussion’ section. Statistical significance was set at two-sided P < 0.05. All analyses were performed using the R statistical software package version 4.2.0 (R Foundation for Statistical Computing).

## Results

**Fig. 1:**
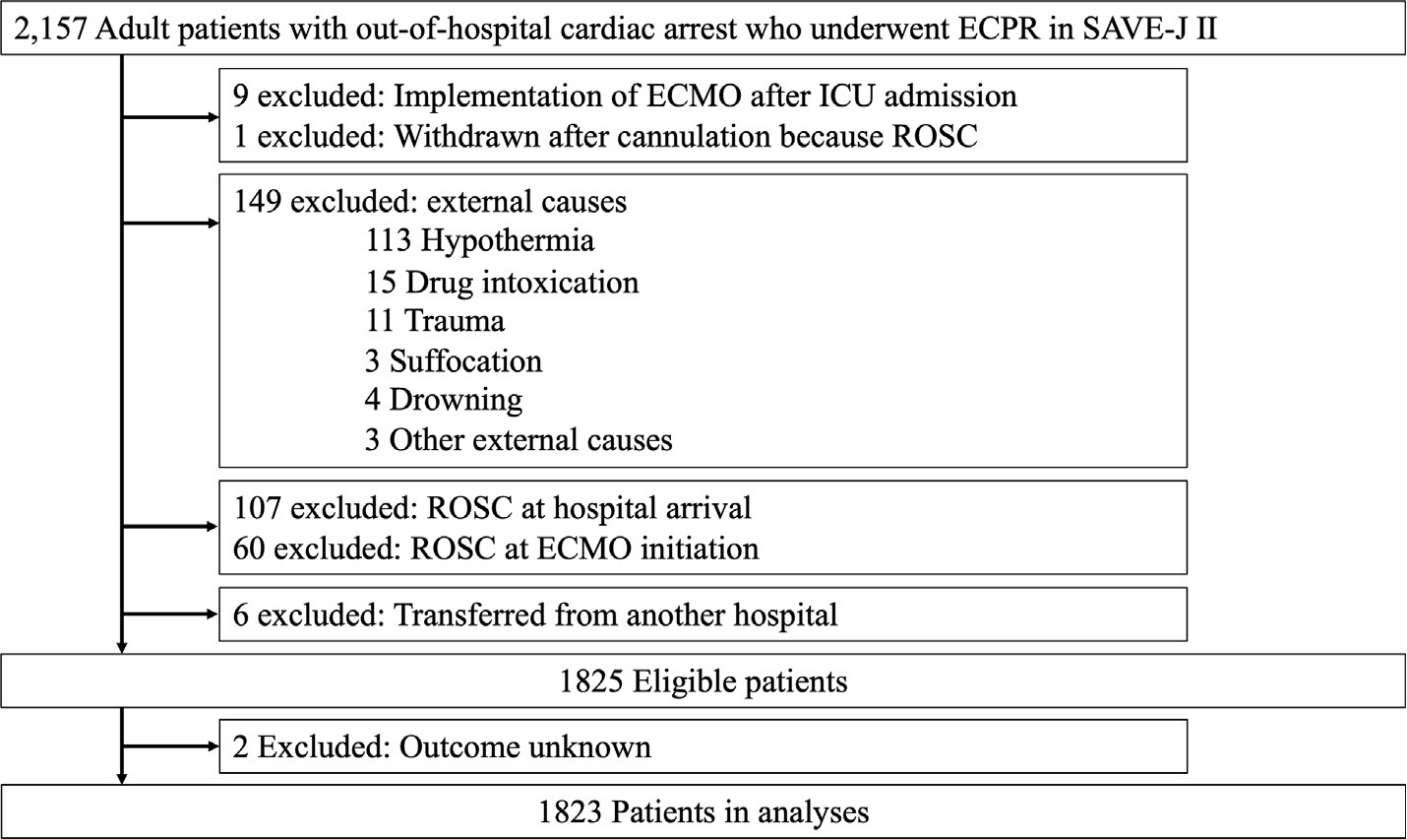
Flowchart for inclusion and exclusion of study participants. ECPR, extracorporeal cardiopulmonary resuscitation. SAVE-J II, Study of Advanced Life Support for Ventricular Fibrillation with Extracorporeal Circulation in Japan. ICU, intensive care unit. ROSC, return of spontaneous circulation.

Between 2013 and 2018, 2157 adult patients with OHCA who received ECPR were enrolled in the SAVE-J II study. Among them, 1823 patients were included in our analysis (Fig. 1).

### Patient characteristics

Table 1 shows a comparison between favourable and unfavourable neurological outcomes for each baseline characteristic of the target population. Shockable cardiac rhythms both at the scene (81.6% vs 61.7%) and upon hospital arrival (73.5% vs 41.9%), and transient ROSC (16.2% vs 8.3%) were significantly more common in the favourable neurological outcome group. Additionally, the favourable neurological outcome group had more gasping (25.6% vs 6.9%), smaller pupil diameter [4.0 (3.0–5.0) mm vs 5.0 (4.0–6.0) mm], and greater pupillary reflex (28.2% vs 6.5%,) upon hospital arrival.

**Table 1:** Baseline characteristics of patients with favorable and unfavorable neurological outcomes at discharge.^a^

	Total n = 1823	Favorable neurological outcome (CPC 1 or 2) n = 234	Unfavorable neurological outcome (CPC 3–5) n = 1589
Age, years	60 (49–60)	56 (45–66)	61 (50–69)
Sex, male	1526 (83.7)	187 (79.9)	1339 (84.3)
Aetiology of arrest			
Cardiogenic	1420 (77.9)	205 (87.6)	1215 (76.5)
Non-cardiogenic	277 (15.2)	19 (8.1)	258 (16.2)
Initial cardiac rhythm at the scene			
Non-shockable	634 (34.8)	40 (17.1)	594 (37.4)
Shockable	1172 (64.3)	191 (81.6)	981 (61.7)
Witnessed cardiac arrest	1432 (78.6)	200 (85.5)	1232 (77.5)
Bystander CPR	1044 (57.3)	165 (70.5)	879 (55.3)
Transient ROSC before hospital arrival	170 (9.3)	38 (16.2)	132 (8.3)
Estimated low-flow time^b^, min	55 (45–67)	51 (41–62)	55 (46–67)
Cardiac rhythm on hospital arrival			
Non-shockable	980 (53.8)	62 (26.5)	918 (57.8)
Shockable	838 (46.0)	172 (73.5)	666 (41.9)
Body movement during resuscitation	24 (1.3)	13 (5.6)	11 (0.7)
Gasping on arrival	170 (9.3)	60 (25.6)	110 (6.9)
Pupil diameter on arrival, mm	5.0 (4.0–6.0)	4.0 (3.0–5.0)	5.0 (4.0–6.0)
Pupillary reflex on arrival, positive	169 (9.3)	66 (28.2)	103 (6.5)
Blood gas on arrival			
pH	6.93 (6.81–7.04)	6.96 (6.83–7.09)	6.92 (6.81–7.03)
PaCO_2_, Torr	67.4 (48.6–88.5)	56.3 (44.6–71.4)	69.5 (49.5–90.0)
PaO_2_, Torr	82.0 (34.8–289.0)	136.7 (47.1–327.1)	79.5 (34.0–278.0)
Lactate, mg/dL	117.0 (91.1–144.0)	120.6 (87.4–144.2)	116.1 (91.8–143.0)
Hb, g/dL	12.8 (10.9–14.6)	13.5 (11.7–15.4)	12.7 (10.8–14.5)

ROSC, Return of spontaneous Circulation; CPR, cardiopulmonary resuscitation; Hb, hemoglobin.

Missing data: Age = 1, Sex = 0, Aetiology of arrest = 126, Initial cardiac rhythm at the scene = 17, Witnessed = 6, Bystander CPR = 28, Transient ROSC = 28, Estimated low flow time = 108, Cardiac rhythm on hospital arrival = 5, Body movement during resuscitation = 18, Gasping on arrival = 214, Pupil diameter on arrival = 221, Pupillary reflex on arrival = 240, pH = 99, PaCO_2_ = 102, PaO_2_ = 110, Lactate = 212, Hb = 64.

a Data are presented as median (quartiles) for continuous variables and as N (percentage) for categorical variables.

b The definition of estimated low-flow time depended on the location of the cardiac arrest. If the cardiac arrest occurred in the ambulance, low-flow time was defined as the time from cardiac arrest to ECMO initiation. If the cardiac arrest occurred anywhere other than an ambulance, low-flow time was defined as the time from the emergency medical services call to ECMO initiation.

### Clinical outcome data

In Table 2, we assessed the impact of differing neurological outcomes on clinical outcome data. The favourable neurological outcome group comprised 234 patients. The total ICU stay was 3 (1–10) days and was longer in the favourable group [11 (8–16) vs 2 (1–7) days, P < 0.001]. The total length of hospital stay was 3 (1–17) days and was longer in the favourable group [35 (22–53) vs 2 (1–8) days, P < 0.001]. The overall in-hospital mortality rate was 75.0%.

**Table 2:** Clinical outcomes of patients with favourable and unfavourable neurological outcomes at discharge.^a^

	Total n = 1823	Favourable neurological outcome (CPC 1 or 2)n = 234	Unfavourable neurological outcome (CPC 3–5) n = 1589	P value
Length of intensive care unit stay, days	3 (1–10)	11 (8–16)	2 (1–7)	<0.001
Length of intensive care unit stay among survivors, days	12 (9–17)	11 (8–16)	14 (10–19)	<0.001
Length of hospital stays, day	3 (1–17)	35 (22–53)	2 (1–8)	<0.001
Length of hospital stay among survivors, days	36 (22–56)	35 (22–53)	37 (22–58)	0.321
In-hospital mortality rate	1368 (75.0)	0 (0)	1368 (87.1)	<0.001

a Data are presented as median (quartiles) for continuous variables and as N (percentage) for categorical variables.

### Logistic regression analysis for favourable neurological outcomes at hospital discharge

We conducted univariate and multivariate logistic regression analyses to examine the association between clinical factors and favourable neurological outcomes (Table 3). Multivariable analysis revealed that shockable rhythm at the scene [odds ratio (OR); 2.11; 95% confidence interval (CI), 1.16–3.95] and upon hospital arrival (OR 2.59; 95% CI 1.60–4.30), bystander CPR (OR 1.63; 95% CI 1.03–1.88), body movement during resuscitation (OR 7.10; 95% CI 1.79–32.90), gasping (OR 4.33; 95% CI 2.57–7.28), pupillary reflex on arrival (OR 2.93; 95% CI 1.73–4.95), and male sex (OR 0.43; 95% CI 0.24–0.75) significantly correlated with neurological outcomes.

**Table 3:** Logistic regression analysis for favorable neurological outcomes at discharge.

	Univariate analysis			Multivariable analysis		
	OR	(95%CI)	P value	OR	(95%CI)	P value
Age, years	0.97	(0.97–0.98)	<0.001	0.97	(0.95–0.98)	<0.001
Sex, male	0.74	(0.53–1.06)	0.09	0.43	(0.24–0.75)	0.002
Aetiology of arrest						
Cardiogenic	Reference			Reference		
Non-cardiogenic	0.44	(0.26–0.69)	<0.001	1.03	(0.46–2.16)	0.95
Initial cardiac rhythm at the scene						
Non-shockable	Reference			Reference		
Shockable	2.89	(2.05–4.18)	<0.001	2.11	(1.16–3.95)	0.017
Witnessed cardiac arrest	1.85	(1.27–2.80)	0.002	1.04	(0.59–1.88)	0.90
Bystander CPR	1.98	(1.47–2.70)	<0.001	1.63	(1.03–1.88)	0.039
Transient ROSC	2.15	(1.44–3.15)	<0.001	1.67	(0.86–3.16)	0.12
Estimated low-flow time^a^, min	0.99	(0.98–0.99)	0.002	0.99	(0.98–1.00)	0.10
Cardiac rhythm on hospital arrival						
Non-shockable	Reference			Reference		
Shockable	3.82	(2.83–5.23)	<0.001	2.59	(1.60–4.30)	<0.001
Body movement during resuscitation	8.42	(3.72–19.4)	<0.001	7.10	(1.79–32.90)	0.007
Gasping on arrival	4.52	(3.15–6.43)	<0.001	4.33	(2.57–7.28)	<0.001
Pupil diameter on arrival, mm	0.66	(0.59–0.74)	<0.001	0.76	(0.65–0.89)	<0.001
Pupillary reflex on arrival, positive	6.22	(4.35–8.89)	<0.001	2.93	(1.73–4.95)	<0.001
Blood gas on arrival						
pH	3.43	(1.55–7.58)	<0.001	0.25	(0.06–1.06)	0.059
PaCO_2_, Torr	0.99	(0.98–0.99)	<0.001	0.98	(0.97–0.99)	<0.001
PaO_2_, Torr	1.00	(1.00–1.00)	0.017	1.00	(1.00–1.00)	0.84
Lactate, mg/dL	1.00	(1.00–1.00)	0.92	1.00	(0.99–1.00)	0.25
Hb, g/dL	1.15	(1.09–1.21)	<0.001	1.21	(1.10–1.34)	<0.001

OR, odds ratio; CI, confidence interval; ROSC, return of spontaneous circulation; CPR, cardiopulmonary resuscitation; GCS-MS, Glasgow Coma Scale-motor scale; Hb, hemoglobin.

a The definition of estimated low-flow time depended on the location of the cardiac arrest. If the cardiac arrest occurred in the ambulance, low-flow time was defined as the time from cardiac arrest to ECMO initiation. If the cardiac arrest occurred anywhere other than an ambulance, low-flow time was defined as the time from the emergency medical services call to ECMO initiation.

## Discussion

In this retrospective cohort study, we showed an association between favourable neurological outcomes and medical information obtained at the time of hospital arrival. The novelty of this study lies in the comprehensive assessment of previously identified prognosis predictors, utilizing a large dataset to evaluate the extent of their impact on neurological outcomes. The factors positively associated with favourable neurological outcomes included shockable rhythm at the scene and on hospital arrival, bystander CPR, body movement during resuscitation, gasping, and pupillary reflex on arrival. The factor negatively associated with favourable neurological outcomes includes being male. Notably, the odds ratios for body movement during resuscitation, gasping, and pupillary reflex on arrival were high; however, these occurrences were not frequent, ranging only from 1.3% to 9.3%.

Our study population differed in several aspects from the inclusion criteria of three previous randomised controlled trials on ECPR.^5^, ^6^, ^7^ This study included fewer witnessed events (78.6%), a lower incidence of bystander CPR (57.3%), and a higher prevalence of non-shockable rhythms at the scene (34.8%) compared to the same parameters in the aforementioned trials. Consequently, favourable neurological outcomes were observed in only 12.8% of all cases in this study, which was lower than those reported in randomised controlled trials using more stringent inclusion criteria (20–40%).^5^, ^6^, ^7^ However, this study found that not only shockable rhythms but also signs of life, including body movement during resuscitation, gasping, and pupillary reflex, correlated with favourable outcomes. The incidence of these signs of life was relatively low, ranging from 1.3% to 9.3%. Despite their infrequency, incorporating these indicators into the inclusion criteria could have enhanced the rate of favourable neurological outcomes.

This study showed that shockable rhythms, body movement during resuscitation, gasping, and pupillary reflex have particularly high odds ratios among the information available from hospital arrival to ECMO-initiation. We found that prehospital shockable rhythm is a useful predictive factor associated with favourable neurological outcomes. Furthermore, the appearance of shockable rhythms at any given time points between cardiac arrest and ECMO initiation may correlate with neurological outcomes. A recent randomised controlled trial show that gasping during CPR is associated with long-term survival and favourable neurological prognosis.^25^ In addition, gasping during transportation is associated with a favourable neurological prognosis in patients with OHCA receiving ECPR.^26^ Thus, gasping is thought to indicate the maintenance of cerebral blood flow and partial functioning of the brain. Combined with shockable rhythms, gasping was shown to be an important natural biomarker predictive of favourable neurological outcomes in cardiac arrest.^25^ The presence or absence of a pupillary reflex during and after resuscitation has been shown to have prognostic value.^27^ The pupillary reflex is one of the most sensitive reflexes in the human body and a direct indicator of brainstem activity.^27^ Recent research has suggested the usefulness of indicators called the signs of life.^15^, ^20^ “Signs of life” is a collective term for body movement during resuscitation, gasping, and pupillary reflex.^20^ Thus, combining several physical findings may yield a system that predicts even more favourable outcomes.

Recently, a systematic review reported on prognostic factors associated with favourable neurological outcomes in patients treated with ECPR for OHCA.^28^ This study included 29 observational and randomised studies involving 7,397 patients.^28^ While our findings regarding factors significantly associated with favourable neurological outcomes, such as sex, shockable rhythm, and bystander CPR, conform with those reported in the review, the novelty of our study lay in its inclusion of additional factors related to the signs of life such as body movement during resuscitation, gasping, and pupillary reflex. Our findings support the decision to administer ECPR to appropriately selected patients and warrant further research. This study concentrated on the potential benefits of implementing aggressive inclusion criteria, rather than exclusion criteria. We do not recommend withholding ECPR in cases where factors crucial for positive outcomes are absent.

### Limitations

Our study has some limitations. First, this was a retrospective observational study, which entailed an inherent potential for selection and recall biases. Second, we did not establish any criteria for introducing ECPR and left it to the discretion of the physicians at each participating facility. Therefore, patients predicted to have poor outcomes may have been excluded from the study. Third, there was no control group (i.e., a patient group that did not receive ECPR). Thus, we examined patients who underwent ECPR in this study despite not knowing if ECPR improved patient prognosis. Fourth, no long-term data on neurological outcomes were available for our analyses. Fifth, no temporal confounding factors, such as the number of ROSCs or total ROSC time until ECMO-initiation, were obtained from this dataset. Therefore, the quality of the transient ROSC data could not be evaluated. Furthermore, due to the lack of different datasets, the estimated low-flow time may differ from the actual low-flow time, especially in patients experiencing transient ROSC. Sixth, the characteristics of our study differed from those of a general ECMO cohort. In our cohort, 34.8% of patients initially presented with a non-shockable rhythm, only 61.2% were defibrillated before hospital arrival, and only 57.3% received bystander CPR. Consequently, these differences may affect the generalisability and external validity of our results. Seventh, missing data may introduce bias into our results. The impact of this bias on our results is uncertain. Finally, the presumed cardiogenic and noncardiogenic aetiologies of cardiac arrest might have been inaccurately diagnosed by physicians.

## Conclusions

This retrospective multicentre cohort study suggests that shockable rhythm, bystander CPR, body movement during resuscitation, gasping, pupillary reflex, and sex were associated with favourable neurological outcomes in patients with OHCA treated with ECPR. Our findings may support the patient selection to administer ECPR. Prospective studies are warranted to validate the findings of the present study.

## CRediT authorship contribution statement

**Naoki Tominaga:** Writing – original draft, Methodology, Investigation, Formal analysis, Conceptualization. **Toru Takiguchi:** Writing – review & editing, Methodology, Investigation, Conceptualization. **Tomohisa Seki:** Writing – review & editing, Methodology, Formal analysis. **Takuro Hamaguchi:** Writing – review & editing, Methodology, Investigation. **Jun Nakata:** Writing – review & editing, Investigation. **Takeshi Yamamoto:** Writing – review & editing, Supervision, Methodology. **Takashi Tagami:** Writing – review & editing, Supervision, Methodology. **Akihiko Inoue:** Project administration. **Toru Hifumi:** Writing – review & editing, Methodology, Investigation, Conceptualization. **Tetsuya Sakamoto:** Project administration. **Yasuhiro Kuroda:** Project administration. **Shoji Yokobori:** Writing – review & editing, Supervision, Project administration, Conceptualization.

## Declaration of competing interest

The authors declare that they have no known competing financial interests or personal relationships that could have appeared to influence the work reported in this paper.
